# Anti-integrin αvβ6 IgG antibody as a diagnostic and prognostic marker in ulcerative colitis: A cross-sectional and longitudinal study defining a specific disease phenotype

**DOI:** 10.1093/ecco-jcc/jjaf062

**Published:** 2025-04-19

**Authors:** Eleftheria Pertsinidou, Benita Salomon, Daniel Bergemalm, Samira Salihovic, Charlotte R H Hedin, Maria Ling Lundström, Åsa V Keita, Maria K Magnusson, Carl Eriksson, May-Bente Bengtson, Olle Grännö, Tone B Aabrekk, Robert Movérare, Niclas Rydell, Helena Ekoff, Johan Rönnelid, BIO-IBD consortium, Sven Almer, Hans Strid, Henrik Hjortswang, Francesca Bresso, Johann Hreinsson, André Blomberg, Adam Carstens, Mauro D’Amato, Trond E Detlie, Gert Huppertz-Hauss, Randi Opheim, Petr Ricanek, Vendel A Kristensen, Lena Öhman, Johan D Söderholm, Robert Kruse, Carl M Lindqvist, Marie Carlson, Dirk Repsilber, Marte L Høivik, Jonas Halfvarson

**Affiliations:** Department of Immunology, Genetics, and Pathology, Uppsala University, Uppsala, Sweden; Thermo Fisher Scientific, Uppsala, Sweden; School of Medical Sciences, Faculty of Medicine and Health, Örebro University, Örebro, Sweden; Department of Gastroenterology, Faculty of Medicine and Health, Örebro University, Örebro, Sweden; School of Medical Sciences, Faculty of Medicine and Health, Örebro University, Örebro, Sweden; Department of Medicine Solna, Karolinska Institutet, Stockholm, Sweden; Department of Gastroenterology, Dermatovenereology and Rheumatology, Centre for Digestive Health, Karolinska University Hospital, Stockholm, Sweden; Department of Medical Sciences: Gastroenterology and Hepatology, Uppsala University, Uppsala, Sweden; Department of Biomedical and Clinical Sciences, Linköping University, Linköping, Sweden; Department of Microbiology and Immunology, Institute of Biomedicine, Sahlgrenska Academy, University of Gothenburg, Gothenburg, Sweden; Department of Gastroenterology, Faculty of Medicine and Health, Örebro University, Örebro, Sweden; Department of Gastroenterology, Vestfold Hospital Trust, Tønsberg, Norway; Department of Laboratory Medicine, Clinical Microbiology, Faculty of Medicine and Health, Örebro University, Örebro, Sweden; Department of Gastroenterology, Vestfold Hospital Trust, Tønsberg, Norway; Institute of Clinical Medicine, University of Oslo, Oslo, Norway; Thermo Fisher Scientific, Uppsala, Sweden; Department of Medical Sciences: Respiratory, Allergy and Sleep Research, Uppsala University, Uppsala, Sweden; Thermo Fisher Scientific, Uppsala, Sweden; Thermo Fisher Scientific, Uppsala, Sweden; Department of Medical Sciences: Gastroenterology and Hepatology, Uppsala University, Uppsala, Sweden; Department of Immunology, Genetics, and Pathology, Uppsala University, Uppsala, Sweden; Gastrointestinal Genetics Lab, CIC bioGUNE—BRTA, Derio, Spain; Ikerbasque, Basque Foundation for Science, Bilbao, Spain; Department of Medicine and Surgery, LUM University, Casamassima, Italy; Institute of Clinical Medicine, University of Oslo, Oslo, Norway; Department of Gastroenterology, Akershus University Hospital, Lørenskog, Norway; Institute of Health and Society, University of Oslo, Oslo, Norway; Institute of Health and Society, University of Oslo, Oslo, Norway; Department of Gastroenterology, Oslo University Hospital, Oslo, Norway; Department of Gastroenterology, Akershus University Hospital, Lørenskog, Norway; Department of Gastroenterology, Lovisenberg Diaconal Hospital, Oslo, Norway; Department of Gastroenterology, Oslo University Hospital, Oslo, Norway; Unger-Vetlesen Institute, Lovisenberg Diaconal Hospital, Oslo, Norway; Department of Microbiology and Immunology, Institute of Biomedicine, Sahlgrenska Academy, University of Gothenburg, Gothenburg, Sweden; Department of Biomedical and Clinical Sciences, Linköping University, Linköping, Sweden; School of Medical Sciences, Faculty of Medicine and Health, Örebro University, Örebro, Sweden; School of Medical Sciences, Faculty of Medicine and Health, Örebro University, Örebro, Sweden; Department of Medical Sciences: Gastroenterology and Hepatology, Uppsala University, Uppsala, Sweden; School of Medical Sciences, Faculty of Medicine and Health, Örebro University, Örebro, Sweden; Institute of Clinical Medicine, University of Oslo, Oslo, Norway; Department of Gastroenterology, Oslo University Hospital, Oslo, Norway; Department of Gastroenterology, Faculty of Medicine and Health, Örebro University, Örebro, Sweden

**Keywords:** inflammatory bowel disease, ulcerative colitis, autoantibodies

## Abstract

**Background and Aims:**

The diagnostic and prognostic properties of anti-integrin αvβ6 immunoglobulin G (IgG) autoantibodies in ulcerative colitis (UC) are poorly understood. We aimed to assess the diagnostic performance of anti-integrin αvβ6 autoantibodies and examine their association with disease outcomes.

**Methods:**

Serum samples from a Swedish inception cohort of patients with suspected inflammatory bowel disease (IBD, *n* = 473) were analyzed using an in-house fluorescence enzyme immunoassay based on EliA technology. Findings were validated in a Norwegian population-based inception cohort (*n* = 570). Diagnostic performance was assessed by calculating the area under the curve (AUC) with 95% confidence intervals and determining sensitivity and specificity. Reclassification was evaluated using the net reclassification index.

**Results:**

In the discovery cohort, patients with UC, IBD-unclassified, or colonic Crohn’s disease exhibited higher median autoantibody levels compared to symptomatic and healthy controls. In the validation cohort, the autoantibody demonstrated 79% sensitivity and 94% specificity for UC vs symptomatic controls at a cut-off of 400 U_A_/l. Its diagnostic performance (AUC = 0.92, 95% CI, 0.89-0.95) was superior to hs-CRP (AUC = 0.65, 95% CI, 0.60-0.70, *P* < .001) and faecal calprotectin (fcalpro) (AUC = 0.88, 95% CI, 0.84-0.92, *P* = .09). Combining the autoantibody with fcalpro further improved diagnostic accuracy (AUC = 0.97, 95% CI, 0.95-0.98) and patient reclassification (*P* < .001). Autoantibody positivity was associated with a severe phenotype of UC, characterised by increased inflammatory activity and higher IL-17A and granzyme B levels. Higher autoantibody levels were linked to an aggressive disease course, remaining stable in aggressive UC but decreasing in indolent disease (*P = *.003).

**Conclusions:**

Anti-integrin αvβ6 is a reliable diagnostic and prognostic marker for UC, with potential clinical implementation.

## Graphical Abstract

**Graphical Abstract:**
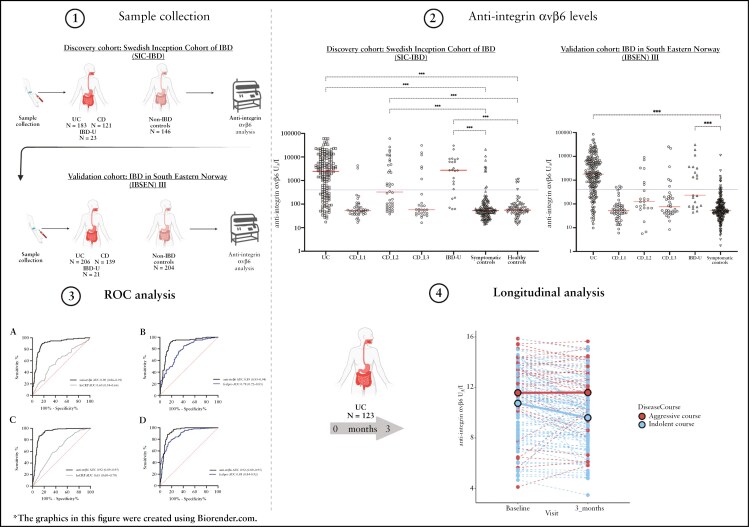

## 1. Introduction

Inflammatory bowel disease (IBD) is a progressive condition characterised by chronic gastrointestinal inflammation. The disease entity comprises the subtypes Crohn’s disease (CD), ulcerative colitis (UC), and IBD-unclassified (IBD-U), the latter being used for patients in whom no further distinction can be made.^1^ The pathophysiology of IBD is marked by significant heterogeneity manifesting in pronounced differences in clinical presentation, endoscopic appearance, and histological features between its subtypes. Important differences in the anatomic distribution of inflammation and disease severity also exist within CD and UC.^2^ However, recent studies have highlighted that patients with UC and those with colonic CD have several molecular characteristics in common and that these features differ from patients with ileal CD.^3–6^ These distinctions advocate for a revised classification system that reflects the evolving understanding of IBD heterogeneity.^7,8^

While autoantibodies play a critical role in diagnosis, patient classification, and stratification in many other chronic inflammatory diseases,^9^ they have not been widely incorporated into diagnostic algorithms or management strategies for IBD.^2^ Recently, a novel autoantibody of immunoglobulin G (IgG) isotype against integrin αvβ6 (anti-integrin αvβ6) was identified in the serum of adult patients with UC in Japan.^10^ Since the initial report, increased levels of anti-integrin αvβ6 have been observed in a small Swedish cohort of adult patients, a Japanese pediatric cohort, and in a North American study,^11–13^ the latter including individuals from a Department of Defense preclinical cohort (PREDICTS) who later in life were diagnosed with UC.^11^ The observed associations may harbor important clues to the pathogenesis of the UC, as integrin αvβ6 has been linked to maintaining epithelial barrier integrity and reducing epithelial inflammation.^14–16^

Collectively, these results indicate that anti-integrin αvβ6 could be relevant in defining UC and its preclinical stages, and potentially also various phenotypes of the disease as an association with a composite of adverse UC-related outcomes have been observed in the North American study. However, interpreting previous studies is challenged by their cross-sectional design, lack of detailed clinical information, and assessment of patients exposed to various treatments or absence of validation in external cohorts. Advancing findings from prior microtiter plate-based enzyme-linked immunosorbent assay (ELISA) to implementation in clinical practice would also facilitate assay development for high-throughput analyses.

Based on these considerations, we examined the association of anti-integrin αvβ6 autoantibodies with different clinical phenotypes at IBD diagnosis in 2 independent inception cohorts of patients with newly diagnosed IBD, using a novel automated high throughput in-house fluorescence enzyme immunoassay (FEIA). We specifically evaluated the diagnostic and prognostic properties of anti-integrin αvβ6 in UC. Additionally, we assessed its utility in defining inflammatory subgroups of UC, explored its association with immuno-inflammatory serum proteins in UC, and ascertained its temporal dynamics in relation to disease course.

## 2. Materials and methods

### 2.1. Study design

**Figure 1.**
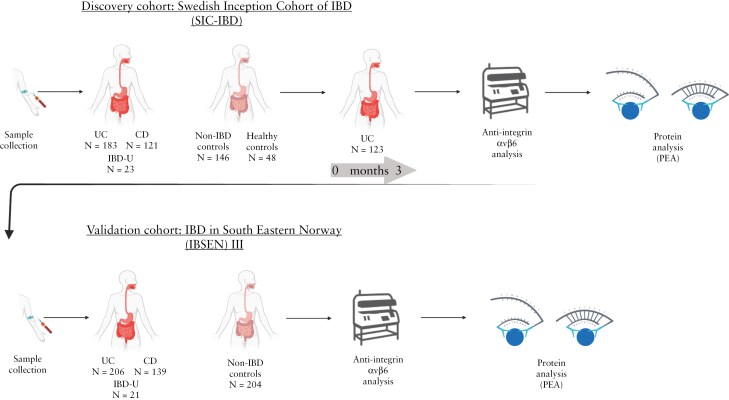
The overall study design. Illustration of the collection of blood samples from a Swedish inception cohort of adult patients with suspected IBD. The study findings were validated using the independent Norwegian population-based IBSEN III inception cohort. The graphics in this figure were created using Biorender.com. Abbreviations: IBD, inflammatory bowel disease; UC, ulcerative colitis; CD, Crohn’s disease; IBD-U, IBD-unclassified.

This was a multi-center cohort study where we analysed anti-integrin αvβ6 IgG in serum samples from the Swedish Inception Cohort of IBD (SIC-IBD), a prospective cohort of adult patients with a clinical suspicion of IBD. Patients diagnosed with UC, CD, and IBD-U and those with symptoms indicative of IBD but who were ultimately determined to be disease-free (ie, symptomatic controls) and healthy controls were compared. Diagnostic and prognostic performance was assessed, and associations between the occurrence of autoantibodies and different clinical phenotypes and serum proteome were examined. In addition, the dynamics of the autoantibody over time were related to the disease course. Findings were validated in serum samples from an independent population-based inception cohort, ie, the IBD in South-Eastern Norway (IBSEN) III cohort, and the diagnostic performance of anti-integrin αvβ6 IgG autoantibodies were compared to existing biomarkers, ie, high-sensitivity C-reactive protein (hs-CRP) and faecal calprotectin (fcalpro). Representatives of the Swedish and Norwegian national patient organizations participated in developing the study and its design (Figure 1).

### 2.2. Patient cohorts

#### 2.2.1. Discovery cohort

Patients aged ≥ 18 years who, due to the suspicion of IBD, were referred to gastroenterology units at 8 hospitals in Sweden between November 2011 and March 2021 were prospectively invited to participate in the SIC-IBD. The diagnosis of IBD was confirmed according to internationally accepted criteria,^2^ following thorough clinical, microbiological, endoscopic, histological, and radiological evaluation. Patients who did not meet the diagnostic criteria for IBD were included as non-IBD symptomatic controls. This group comprised patients ultimately diagnosed with diseases such as microscopic colitis, infectious enteritis, celiac disease, and irritable bowel syndrome. In addition, healthy individuals without a history of chronic gastrointestinal disease or symptoms were separately recruited as a second control group.

#### 2.2.2. Validation cohort

To validate the biological relevance of the findings in the discovery cohort, we used treatment-naïve patients aged ≥ 18 years from IBSEN III, a population-based inception cohort. All patients were recruited within the Norwegian South-Eastern Health Region, Norway, from January 2017 to December 2019. As described elsewhere, a uniform approach was employed for clinical work-up, diagnostic criteria, and classification systems.^17^ Symptomatic controls comprised individuals referred to the gastroenterologist with suspected IBD but who had normal endoscopic findings and no evidence of IBD upon diagnostic work-up. Further details about the 2 cohorts are provided in the Supplementary Materials.

### 2.3. Disease course outcome during follow-up

A composite outcome measure defined the disease course within the first year of diagnosis as aggressive or indolent. An aggressive course of UC was defined as colectomy, hospital admission for active disease, unresponsiveness to ≥ 2 advanced therapies (ie, biologics or JAK inhibitors), or extensive use of corticosteroids. The latter was defined as the use of > 2 courses of corticosteroids or a cumulative corticosteroid dose of > 2.5 g equivalents of prednisolone within the first year. Besides the criteria mentioned above, the definition of an aggressive course of CD also included evidence of progression to a complicated disease behavior (ie, a new stricture, fistula, or abscess) and surgical procedures related to these complications.

### 2.4. Ethical considerations

All participants provided written informed consent, and the study was conducted according to the Declaration of Helsinki. Ethical permission was granted by the Regional Ethics Review Boards, the SIC-IBD cohort (Dnr. 2010/313), and the IBSEN III cohort (2015/946).

### 2.5. Anti-integrin αvβ6 IgG autoantibodies

Serum IgG autoantibodies against integrin αvβ6 were measured using a novel in-house research FEIA based on EliA technology (Thermo Fisher Scientific/Phadia AB, Uppsala, Sweden). The assay using generic standard EliA reagents was adapted from a previously described ELISA.^13^ Serum samples were diluted 1:100 prior to analysis. The assay runs were performed using an automated Phadia 250 instrument (Thermo Fisher Scientific/Phadia AB) that converted the sample assay response units to anti-integrin αvβ6 serum concentrations expressed as arbitrary units per liter (U_A_/l).^18^

### 2.6. High-sensitivity C-reactive protein

After recruitment, hs-CRP was assayed in a single batch for each cohort. Concentrations were measured with a particle-enhanced immunoturbidimetric hs-CRP assay (Cardiac C-Reactive Protein [Latex] High Sensitive, Roche Diagnostics, Rotkreuz, Switzerland) on a Roche Cobas c501 at Uppsala BioLab, Uppsala Clinical Research Centre, Uppsala, Sweden.

### 2.7. Faecal calprotectin

In the discovery cohort, faecal samples were extracted and analysed according to the manufacturer’s instructions using a chemiluminescent immunoassay and the LIASON XL analyser (DiaSorin, Saluggia, Italy). For the validation cohort, concentrations of faecal calprotectin were measured using the Bühlmann calprotectin ELISA EK-CAL (Bühlmann Laboratories, Schönenbuch, Switzerland) after sample extraction.

### 2.8. Protein analyses

Given the previously suggested role of anti-integrin αvβ6 autoantibodies in mucosal homeostasis,^10^ we hypothesised that the serum proteome of patients with UC would differ based on anti-integrin αvβ6 status. Relative levels of 176 proteins in serum from patients with UC were measured by proximity extension assay (PEA) methodology using the Proseek Multiplex Inflammation I and Proseek Multiplex Oncology II panels (Olink Proteomics, Uppsala, Sweden). Details about the preprocessing of protein data are provided in the Supplementary Material.

### 2.9. Statistical analysis

Continuous variables are presented as the median (interquartile range), while categorical variables are represented as frequencies with corresponding percentages. The Kruskal–Wallis test assessed the difference in median anti-integrin αvβ6 concentrations between all groups. The Dunn’s multiple comparisons test was performed post hoc to identify which pairs of groups were significantly different. For categorical data, a χ^2^ test or Fisher’s exact test (for expected frequencies < 5) was performed; for continuous parameters, the Mann–Whitney *U* test was used. The diagnostic cut-off for positive results was defined as 400 U_A_/l, corresponding to the 96th percentile of 196 healthy individuals. Receiver operating characteristic (ROC) curves and area under the curve (AUC) with 95% confidence intervals (CIs) were generated to evaluate and compare the diagnostic and prognostic performance of different markers using logistic regression. Comparisons between the AUC values were performed using DeLong’s 2-sided test. The Youden index was used to derive the optimal cut-off value for differentiating between an aggressive and an indolent course of UC and CD in the discovery cohort, which was further used to determine sensitivity, specificity, and likelihood ratio for a positive result (LR[+]), and likelihood ratio for a negative result (LR[−]) in the validation cohort. Reclassification was assessed using the net reclassification index (NRI) and integrated discrimination improvement (IDI) index.^19^ To analyse longitudinal data, mixed-effect models were employed, using the lme function in R and a maximum likelihood approach. The model equation incorporated anti-integrin αvβ6 levels as the outcome variable, with fixed effect terms for visit (baseline or 3-month visit), disease course, and their interaction (visit * disease course). A random intercept for individual patients was also included to account for individual baseline levels. A principal component analysis was performed to visualise differences in protein profiles between anti-integrin αvβ6 positive and negative patients. Welch’s *t*-test was used to screen for significantly differing proteins between patients in these 2 groups. The *P*-value of Welch’s *t*-test was adjusted (*q*-value, significant if *q* < 0.05) to control for the false discovery rate (FDR) estimates, which were calculated according to the Benjamini-Hochberg procedure for multiple testing. Correlations between the significant proteins and anti-integrin αvβ6 levels were calculated using the Pearson correlation coefficient.

The statistical calculations were performed using JMP V.17 (SAS Institute, Cary, NC, USA), the statistical computing language STATA V.16 (College Station, TX: StataCorp LLC), and R V.4.05 (R Foundation for Statistical Computing, Vienna, Austria) with packages sva, tidyverse, dplyr, impute, lmerTest, nlme, ggplot2, ggrepel, multtest, cowplot, and Rlabkey. GraphPad Prism V.10 (GraphPad, San Diego, CA, USA) was used for graph generation. *P*-values <.05 were considered statistically significant.

## 3. Results

### 3.1. Characteristics of patients in the discovery and validation cohort

Table 1 presents the demographics and clinical characteristics of the study participants in the 2 inception cohorts. The discovery cohort comprised 327 patients with IBD, 146 symptomatic non-IBD controls, and 48 healthy controls from the SIC-IBD cohort. The validation cohort included 366 patients with IBD and 204 symptomatic controls from the IBSEN III cohort. In both cohorts, patients were included at the date of diagnosis, and 91% (296/327) of the IBD patients in the discovery cohort and all patients in the validation cohort were treatment-naïve. In the validation cohort, the median period (months) from onset of symptoms to diagnosis was 4 (0-240) for UC and 9 (0-480) for CD, whereas this information was not available for the discovery cohort. The median age at diagnosis of IBD was 32 years in the SIC-IBD and 33 years in the IBSEN III cohort, and also the male-to-female ratio was comparable between the 2 cohorts (1.2:1 vs 1.1:1). Information about country of birth was available in 77% (439/570) of patients in the validation cohort and 85% (375/439) of these patients were born in the Nordics, whereas data on ethnicity were not collected for the discovery cohort. While no significant differences were observed in the extent of UC (*P* = .11), the distribution of disease location in patients with CD varied between the 2 cohorts (*P = *.04), as detailed in Table 1.

**Table 1.** Clinical characteristics and demographics of the discovery and validation cohorts.

	Discovery SIC-IBD			Validation IBSEN III		IBD SIC-IBD vs IBD IBSEN III
	IBD (*N* = 327)	Symptomatic controls (*N* = 146)	Healthy controls (*N* = 48)	IBD (*N* = 366)	Symptomatic controls (*N* = 204)	*P*-value
**Sex**						.54
Male, *N* (%)	180 (55)	57 (39)	20 (42)	193 (53)	96 (47)	
**Median age, years (IQR)**	32 (25-43)	34 (24-47)	26 (22-30)	33 (26-47)	30 (25-38)	.22
**Ulcerative colitis, *N* (%)**	183 (56)	NA	NA	206 (56)	NA	.93
**Crohn’s disease, *N* (%)**	121 (37)	NA	NA	139 (38)	NA	.79
**IBD-U, *N* (%)**	23 (7)	NA	NA	21 (6)	NA	.48
**Crohn’s disease location, *N* (%)**						**.04**
L1 Ileal (+/- L4)	42 (38)	NA	NA	54 (49)	NA	
L2 Colonic (+/- L4)	40 (36)	NA	NA	22 (20)	NA	
L3 Ileocolonic (+/- L4)	28 (25)	NA	NA	35 (32)	NA	
L4 Upper gastrointestinal tract	1 (1)	NA	NA	NA	NA	
**Crohn’s disease behaviour, *N* (%)**						.68
B1 Non-stricturing, non-penetrating	95 (83)	NA	NA	89 (80)	NA	
B2 Stricturing	14 (12)	NA	NA	18 (16)	NA	
B3 Penetrating	5 (4)	NA	NA	4 (4)	NA	
**Ulcerative colitis extent, *N* (%)**						.11
E1 Proctitis	54 (31)	NA	NA	66 (39)	NA	
E2 Left-sided colitis	51 (29)	NA	NA	34 (20)	NA	
E3 Extensive colitis	71 (40)	NA	NA	68 (40)	NA	
**Median hs-CRP, g/L (IQR)**	4.5 (1.7-12)	1.7 (0.8-5.9)	0.6 (0.3-1.1)	3.5 (1-10)	1 (0.8-3.2)	**.03**
**Median faecal calprotectin, μg/g (IQR)**	451 (157-1045)	84 (17.4-167)	10.3 (5-26.8)	414 (143.5-1402)	44 (29-104)	.53
**Smoking habits, *N* (%)**						.18
Never smoker	160 (58)	63 (62)	35 (74)	164 (56)	91 (64)	
Former smoker	81 (29)	25 (25)	9 (19)	103 (35)	36 (25)	
Active smoker	36 (13)	13 (13)	3 (6)	27 (9)	16 (11)	

Statistical analyses were conducted using the χ^2^ test or Fisher’s exact test (for expected frequencies < 5) and, for continuous parameters, the Mann–Whitney *U* test. Information was missing in SIC-IBD for: CD location *n* = 10, CD behaviour *n* = 7, UC extent *n* = 7, hs-CRP *n* = 2 (IBD), faecal calprotectin *n* = 78 (IBD), *n* = 41 (symptomatic controls) and *n* = 8 (healthy controls), Smoking habits *n* = 50 (IBD), *n* = 45 (symptomatic controls), *n* = 1 (healthy control); and in IBSEN III for: CD location *n* = 28, CD behaviour *n* = 28, UC extent n = 38, hs-CRP *n* = 3 (IBD) and *n* = 2 (symptomatic controls), faecal calprotectin *n* = 80 (IBD) and *n* = 53 (symptomatic controls), Smoking habits *n* = 72 (IBD) and *n* = 61 (symptomatic controls).

Abbreviations: IBD, inflammatory bowel disease; UC, ulcerative colitis; CD, Crohn’s disease; IBD-U, IBD-unclassified; IQR, interquartile range; hs-CRP, high-sensitivity C-reactive protein; NA, not applicable.

### 3.2. Disease categories by anti-integrin αvβ6 status and levels

**Figure 2.**
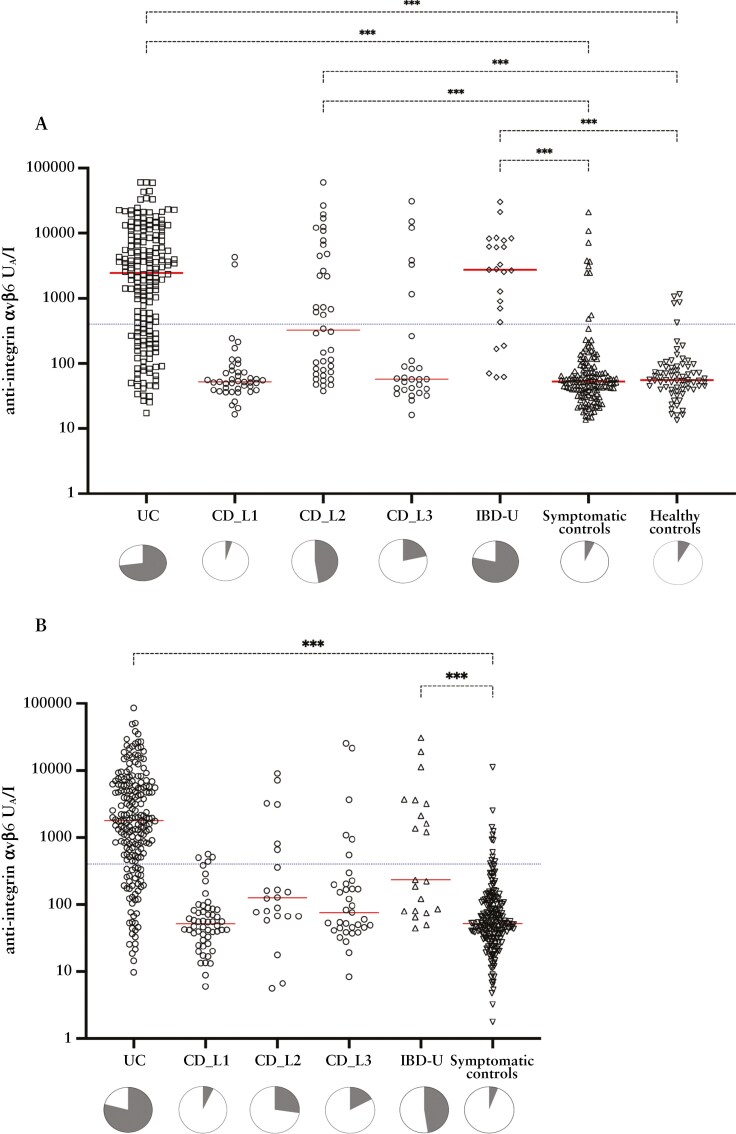
Median levels of anti-integrin αvβ6 differed between disease categories in both the SIC-IBD and IBSEN III cohorts (Kruskal–Wallis *P* < .001, for both cohorts). A) Anti-integrin αvβ6 levels by disease groups in SIC-IBD and B) IBSEN III. The red line represents the median, and the blue dotted line is set to the suggested cut-off (400 U_A_//l). Asterisks show significant *P-*values calculated from Dunn’s multiple comparison test (***P < .001). Pie charts show the proportion of anti-integrin positives (grey) vs negatives (white). Abbreviations: UC, ulcerative colitis; CD, Crohn’s disease; IBD-U, IBD-unclassified.

Median levels of anti-integrin αvβ6 differed significantly between disease categories in the SIC-IBD and IBSEN III cohorts (for both cohorts, Kruskal–Wallis *P* < .001, Figure 2A and B). In the SIC-IBD cohort, higher median anti-integrin αvβ6 autoantibody levels were observed in patients with UC, IBD-U, and colonic CD (L2) compared to symptomatic (*P < *.001) and healthy controls (*P < *.001). In contrast, no differences were seen between patients with ileal CD (L1) or ileocolonic CD (L3) and the 2 control groups. Correspondingly, higher median levels were observed in patients with UC and IBD-U compared to the symptomatic controls in the IBSEN III cohort (*P < *.001). In contrast, the difference between patients with colonic CD (L2) and symptomatic controls was not statistically significant (*P = *.07). The distribution of positivity for anti-integrin αvβ6 autoantibodies for each disease category is depicted in Figure 2A and B; Supplementary Table 1.

**Figure 3.**
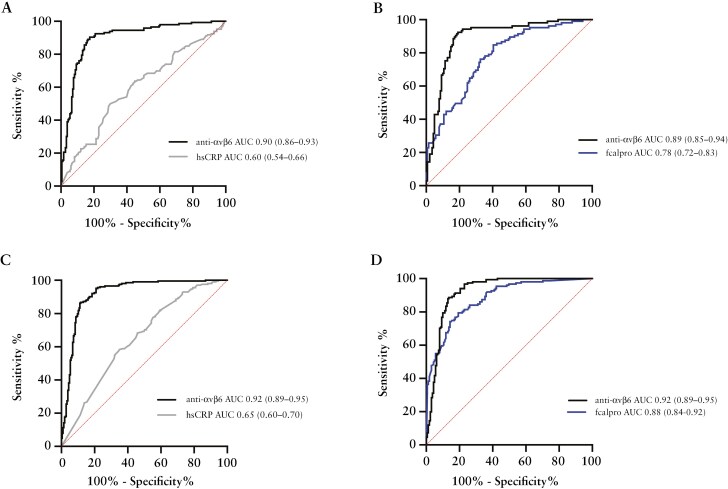
Using logistic regression, the receiver operating characteristic (ROC) curves illustrate the diagnostic prediction of ulcerative colitis vs symptomatic controls in the discovery and validation cohorts. The model performance and validity measures were as follows in the discovery cohort: (A) AUC (95% CI) for anti-integrin αvβ6: 0.90 (0.86-0.93) vs hs-CRP: 0.60 (0.54-0.66), *P < *.001), (B) anti-integrin αvβ6: 0.89 (0.85-0.94) vs fcalpro: 0.78 (0.72-0.83), *P < *.001. In the validation cohort, the corresponding measures were as follows: (C) AUC (95% CI) for anti-integrin αvβ6: 0.92 (0.89-0.95) vs hs-CRP: 0.65 (0.60-0.70), *P < *.001, (D) anti-integrin αvβ6: 0.92 (0.89-0.95) vs fcalpro: 0.88 (0.84-0.92), *P* = .09. Faecal samples were available for 141 patients with UC and 105 symptomatic controls in the Swedish discovery cohort. In comparison, 161 patients with UC and 151 symptomatic controls in the Norwegian validation cohort provided a faecal sample. DeLong’s 2-sided test was used to compare ROC curves. Abbreviations: CI, confidence interval; fcalpro, faecal calprotectin; hs-CRP, high-sensitivity C-reactive protein.

The diagnostic performance of the anti-integrin αvβ6 autoantibody to differentiate UC patients from symptomatic controls in the discovery cohort (AUC = 0.90, 95% CI, 0.86-0.93) was superior to hs-CRP (AUC = 0.60, 95% CI 0.54-0.66), (*P* < .001) (Figure 3A). Additionally, among patients providing a stool sample (N = 246), with missing data for 41 symptomatic controls and 42 UC patients, the diagnostic accuracy of anti-integrin αvβ6 (AUC = 0.89, 95% CI, 0.85-0.94) was significantly higher than fcalpro (AUC = 0.78, 95% CI, 0.72-0.83, *P < *.001) (Figure 3B). The validation cohort also confirmed these findings, showing that anti-integrin αvβ6 had a higher diagnostic performance than hs-CRP (AUC = 0.92, 95% CI, 0.89-0.95 vs AUC = 0.65, 95% CI, 0.60-0.70, *P < *.001) (Figure 3C), and a nominally higher AUC compared to fcalpro (*N* = 312), with missing data for 53 symptomatic controls and 45 UC patients, (AUC = 0.92, 95% CI, 0.89-0.95 vs AUC = 0.88. 95% CI, 0.84-0.92, *P* = .09) (Figure 3D). Adding fcalpro to the anti-integrin αvβ6 model further improved its performance to predict UC in the discovery (AUC = 0.92, 95% CI, 0.89-0.96, *P* = .02) and validation cohort (AUC = 0.97, 95% CI, 0.95-0.98, *P* < .001). Conversely, incorporating hs-CRP in the anti-integrin αvβ6 model did not improve diagnostic accuracy over anti-integrin αvβ6 alone in either the discovery or validation cohort (data not shown).

With a cut-off of 400 U_A_/l, the anti-integrin αvβ6 assay demonstrated a sensitivity of 73% and a specificity of 93% in differentiating UC from symptomatic controls in the discovery cohort. Likelihood ratio for a positive result (LR[+]) was 10.4 and likelihood ratio for a negative result (LR[−]) was 0.3. The corresponding measures in the validation cohort were 79% and 94%, with LR(+) equal to 13.2 and LR(−) equal to 0.22. Applying the same cut-off to compare UC with colonic CD yielded a sensitivity of 73%, a specificity of 55%, LR(+) of 1.6 and LR(−) of 0.5 in the discovery cohort. The corresponding estimates in the validation cohort were 79%, 73%, 2.9, and 0.3.

To examine the clinical relevance of the autoantibody, we evaluated its ability to reclassify patients with UC vs symptomatic controls in both cohorts. The addition of anti-integrin αvβ6 to either hs-CRP or fcalpro significantly improved patient reclassification, as demonstrated by analyses of NRI and IDI in the 2 cohorts (all *P* < .001) (Supplementary Tables 2–5). The autoantibody resulted in a 12% improvement in reclassifying UC cases and a 21% improvement in reclassifying symptomatic controls when combined with fcalpro in the discovery cohort. This combination enhanced both the accuracy and utility of the model. The corresponding improvements in the validation cohort were 11% and 11%. These findings suggest that the autoantibody offers additional clinical value compared to fcalpro alone.

### 3.3. Clinical parameters by anti-integrin αvβ6 status and levels

Based on the observed associations of anti-integrin αvβ6, we examined autoantibody positivity in regard to demographic factors and different clinical phenotypes in patients with UC and colonic CD. However, the number of patients with IBD-U was insufficient for additional analyses of this disease category. No differences in age, sex, or smoking status were observed between anti-integrin αvβ6 positive and negative patients with UC or colonic CD in the 2 cohorts, except for a lower median age at diagnosis of UC in autoantibody-positive patients in the IBSEN III cohort (*P = *.01) (Supplementary Tables 6 and 7).

**Figure 4.**
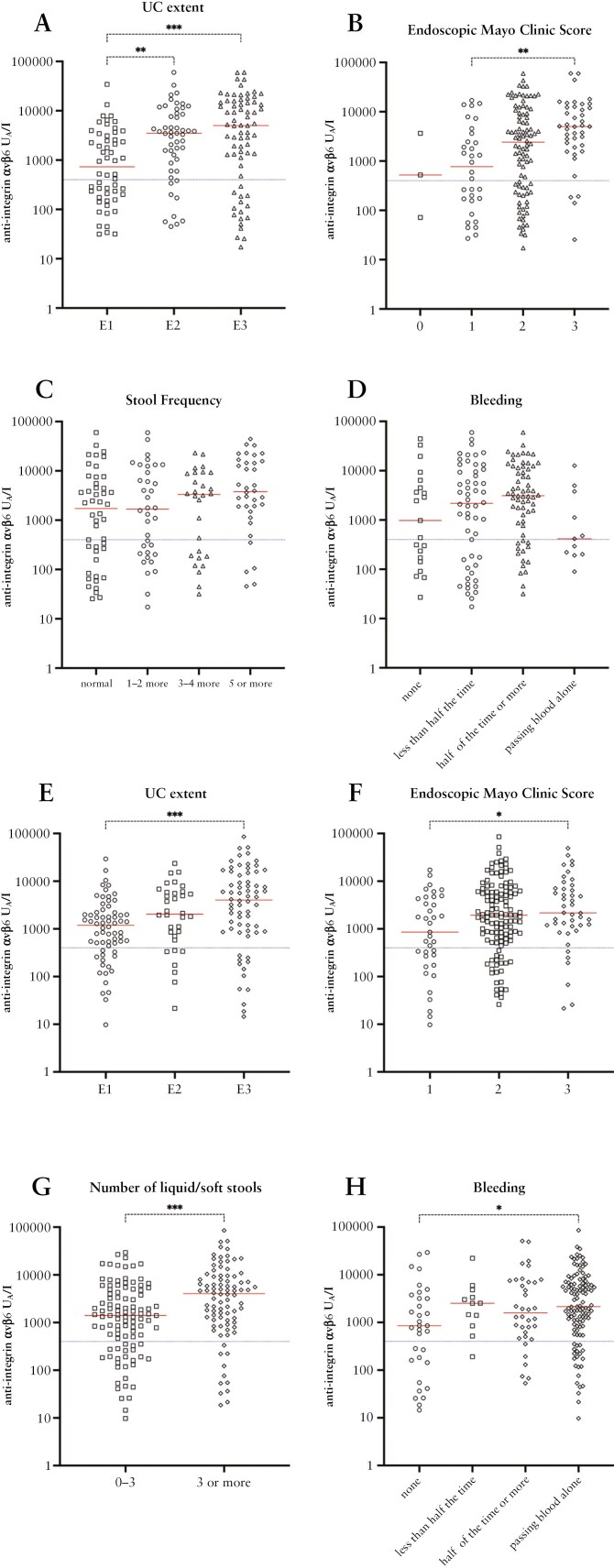
Higher median levels of anti-integrin αvβ6 with increasing extent of UC and endoscopic activity but not with severity of PROs. Anti-integrin αvβ6 levels by A) UC extent, B) Endoscopic Mayo score, C) Stool frequency, D) Bleeding in UC patients in the discovery cohort and E) UC extent, F) Endoscopic Mayo score, G) Number of liquid/soft stools, H) Bleeding in UC patients in the validation cohort. The red line represents the median, and the blue dotted line is set to the suggested cut-off (400 U_A_/l). Asterisks show significant *P*-values as given from Dunn’s multiple comparison test and the Mann–Whitney *U* test (for binary data) (*P < .05, **P < .01, ***P < .001). Abbreviations: UC, ulcerative colitis; PRO, patient-reported outcome.

Overall, anti-integrin αvβ6 autoantibody positivity was associated with a severe phenotype of UC in the discovery cohort, including more extensive disease distribution (*P = *.008), a higher endoscopic Mayo Clinic subscore (*P = *.005), and systemic inflammation, defined as higher hs-CRP (*P = *.04) and lower albumin concentrations (*P = *.01) (Table 2). However, no associations were found with fcalpro levels or the patient-reported outcome (PRO) variables. In line with the results on autoantibody positivity, higher median levels of anti-integrin αvβ6 were observed with a greater extent of UC and endoscopic activity but not with the severity of PROs (Figure 4A–D). The association between anti-integrin αvβ6 and increased inflammation, as shown by the Mayo Clinic endoscopic subscore, was confirmed in UC patients in the validation cohort (Table 2). However, the IBSEN III cohort could not validate the difference in median hs-CRP levels between anti-integrin αvβ6 positive and negative UC patients (*P* = .41). Higher median levels of anti-integrin αvβ6 were observed with increasing disease extent and endoscopic activity in patients with UC in the discovery cohort and were confirmed in the validation cohort (Figure 4A and B, E and F). In addition, higher levels were noted in UC patients with increasing severity of PROs in the IBSEN III cohort (Figure 4G-H). For colonic CD, there was no significant difference between autoantibody positive and negative patients in the discovery (Supplementary Table 8) or validation cohort (Supplementary Table 9) for any of the studied measures.

**Table 2.** Associations of anti-integrin αvβ6 with clinical parameters and patient-reported outcomes in patients with ulcerative colitis in the discovery (*N* = 183) and validation (*N* = 206) cohort.

	Discovery SIC-IBD			Validation IBSEN III		
	Anti-integrin αvβ6 positives	Anti-integrin αvβ6 negatives	*P*-value	Anti-integrin αvβ6 positives	Anti-integrin αvβ6 negatives	*P*-value
UC extent, *N* (%)						
E1 Proctitis	31 (24)	23 (48)	**.008**	50 (38)	16 (46)	.64
E2 Left-sided colitis	42 (33)	9 (19)		27 (20)	7 (20)	
E3 Extensive colitis	55 (43)	16 (33)		56 (42)	12 (34)	
Median (IQR) partial Mayo Clinic score	5 (4-7)	4 (2-6)	**.002**	NA	NA	
Endoscopic Mayo Clinic score, *N* (%)						
0 (remission)	2 (2)	1 (2)	**.005**	NA	NA	**.02**
1 (mild)	18 (15)	14 (30)		23 (14)	14 (33)	
2 (moderate)	64 (53)	28 (60)		102 (63)	23 (53)	
3 (severe)	37 (31)	4 (9)		38 (23)	6 (14)	
Median (IQR) hs-CRP	4 (1.5-9.8)	2.2 (0.8-5.6)	**.04**	2.9 (1-8.4)	2.3 (1-5.1)	.41
Median (IQR) faecal calprotectin (μg/g)	526 (127.5-1417.5)	258.5 (74.3-813.3)	.08	500 (153.3-1801)	373 (117-885)	.22
Median (IQR) albumin (g/L)	38 (34-42)	40 (36.5-43)	**.01**	NA	NA	
Median (IQR) number of liquid/soft stools	NA	NA		3.5 (1-6)	2 (0-4)	**.003**
Stool frequency, N (%)						
Normal	30 (29)	16 (37)	.13	NA	NA	
1-2 more	23 (22)	13 (30)		NA	NA	
3-4 more	19 (18)	9 (21)		NA	NA	
5 or more	31 (30)	5 (12)		NA	NA	
Bleeding, *N* (%)						
None	12 (11)	9 (20)	.16	21 (13)	12 (30)	.08
Less than half the time	38 (36)	16 (36)		11 (7)	1 (3)	
Half of the time or more	51 (48)	14 (32)		30 (19)	6 (15)	
Passing blood alone	6 (6)	5 (11)		94 (60)	21 (53)	

Statistical analyses were conducted using the χ^2^ test or Fisher’s exact test (for expected frequencies < 5) and, for continuous parameters, the Mann–Whitney *U* test. Information was missing in SIC-IBD for UC extent *n* = 7, partial Mayo Clinic score *n* = 46, endoscopic Mayo Clinic score *n* = 15, faecal calprotectin *n* = 42, albumin n = 1, stool frequency *n* = 37, bleeding *n* = 32; and in IBSEN III for: UC extent *n* = 38, CRP *n* = 3, faecal calprotectin *n* = 45, number of liquid/soft stools *n* = 14, Bleeding *n* = 10.

Abbreviations: UC, ulcerative colitis; hs-CRP, high-sensitivity C-reactive protein; IQR, interquartile range; NA, not applicable.

### 3.4. Anti-integrin αvβ6 is associated with a specific set of proteins

To test the hypothesis that the serum proteome differs according to anti-integrin αvβ6 autoantibody status, we analyzed 154 serum proteins from UC patients in the discovery cohort. Principal component analysis (Supplementary Figure 1) did not reveal any distinct separation in the overall protein profile between patients who were positive vs negative for anti-integrin αvβ6.

Next, we examined individual proteins by autoantibody status. Data were used from 173 patients with UC in the SIC-IBD cohort and 201 patients with UC in the IBSEN III cohort. We identified 5 proteins (SYND1, IL-17A, GZMB, MMP1, and CXCL13) that had significantly higher relative levels (after applying a threshold of *q* < 0.05 and a fold change threshold of 1.2, or log2[1.2] on a log2 scale) in anti-integrin αvβ6 positive compared to negative UC patients in the discovery cohort (Supplementary Figure 2A; Supplementary Table 10). The importance of these proteins was validated in IBSEN III. After applying the same test, the upregulation of 2 of the most relevant proteins (Interleukin 17A [IL-17A] and Granzyme B [GZMB]) was confirmed (Supplementary Figure 2B; Supplementary Table 10). To corroborate these findings, we performed correlation analyses between the 2 significantly altered proteins in both cohorts and the anti-integrin αvβ6 levels. For IL-17A, correlations were observed in the discovery cohort (*r* = 0.39, *q* < 0.0001) and validation cohort (*r* = 0.34, *q* < 0.0001) (Supplementary Figure 3). Likewise, significant correlations were observed for GZMB in both the discovery cohort (*r* = 0.33, *q* < 0.001) and the validation cohort (*r* = 0.31, *q* < 0.001).

### 3.5. Anti-integrin αvβ6 as a prognostic biomarker for aggressive disease

**Figure 5.**
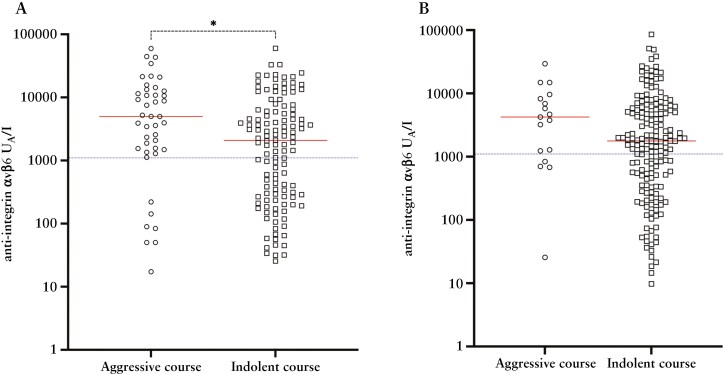
Anti-integrin αvβ6 levels are higher in patients with an aggressive course of UC compared to those with an indolent course. Anti-integrin αvβ6 levels by disease course in UC patients of A) SIC-IBD and B) IBSEN III. The red line represents the median, and the blue dotted line is set to the suggested cut-off (1100 U_A_/l). Asterisks show significant *P*-values from the Mann–Whitney *U* test (*P < .05). The disease course was based on a composite outcome of colectomy, hospital admission for active disease, treatment refractoriness towards ≥ 2 biological agents, and the use of > 2 courses of corticosteroids or a cumulative corticosteroid dose of > 2.5 g.

The prognostic significance of anti-integrin αvβ6 was evaluated in predicting disease course outcomes, defined as aggressive or indolent UC. The logistic regression model for differentiating patients with indolent UC from those with an aggressive course demonstrated an AUC (95% CI) of 0.62 (0.52-0.72) in the discovery cohort (Supplementary Figure 4). Applying this model to the validation cohort yielded an AUC of 0.61 (0.48-0.75), with a sensitivity of 76% and a specificity of 37% at its optimal cut-off (1100 U_A_/l), corresponding to an LR(+) of 1.2 and an LR(−) of 0.6. There was a statistically significant association between autoantibody status (positive vs negative) when applying the prognostic cut-off to the discovery cohort (Supplementary Table 11, *P = *.003). Patients with an aggressive course of UC showed higher anti-integrin αvβ6 levels compared to those with an indolent course in the SIC-IBD cohort (Figure 5A, *P = *.02). Additionally, the proportion of patients with an aggressive disease progression was numerically higher among autoantibody positive (11%) compared to the autoantibody-negative patients (6%) in the validation cohort (Supplementary Table 11, *P* *=* .26). Although not statistically significant, patients with an aggressive disease course showed higher antibody levels compared to those with an indolent disease course in the IBSEN III cohort (Figure 5B, *P* *=* .13).

The performance of the logistic regression model remained unchanged (AUC = 0.61 [0.48-0.75]) in the validation cohort when repeating the analyses and excluding hospital admission from the criteria for aggressive disease course. However, when reiterating the analyses and allowing patients with UC who start at least 1 targeted therapy within the first year of diagnosis to be included in the composite outcome for aggressive disease (all the other criteria remained the same), an AUC of 0.72 (95% CI, 0.62-0.82) was observed (Supplementary Figure 5).

**Figure 6.**
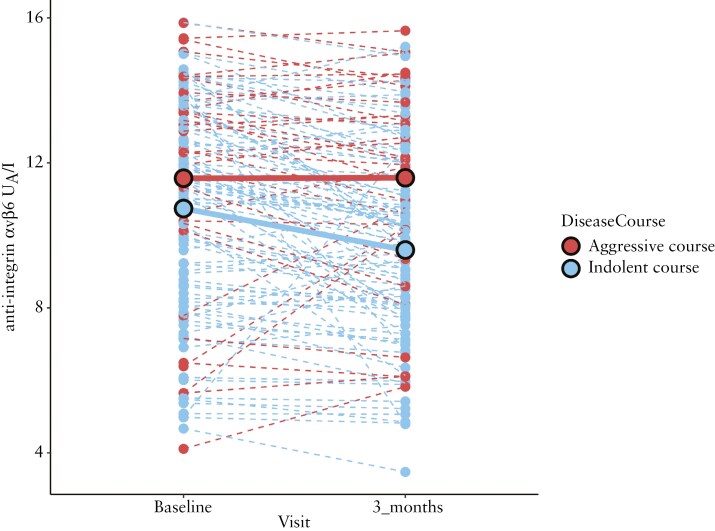
A significant interaction was found between the disease course and visits in UC patients in the discovery cohort. Changes in anti-integrin αvβ6 levels between baseline and 3 months in 123 UC patients in the SIC-IBD cohort. Dark red dotted lines represent the change in anti-integrin αvβ6 levels in patients with an aggressive disease course (*N* = 33) and blue lines represent the change in anti-integrin αvβ6 levels in patients with an indolent course (*N* = 90). Solid lines show the mean change of anti-integrin αvβ6 levels on the log2 scale from baseline to 3 months. For the indolent disease course, the mean log2 change was −1.15; *P* < .001; for the aggressive course, the mean log2 change was 0.01; *P = *.97. Statistical analysis was performed using mixed-effect models. The disease course was based on a composite outcome of colectomy, hospital admission for active disease, treatment refractoriness towards ≥ 2 biological agents, and the use of > 2 courses of corticosteroids or a cumulative corticosteroid dose of > 2.5 g.

Next, we examined longitudinal changes in anti-integrin αvβ6 levels from baseline to 3 months in patients who had serum samples collected at both time points (*N* = 123). The autoantibody levels differed significantly between patients with indolent and aggressive disease courses (*P* *=* .003). More specifically, there was a significant decrease for UC patients with an indolent disease trajectory (mean log2 change −1.15, *P < *.001). In contrast, patients with an aggressive disease had no significant change in their anti-integrin αvβ6 levels (mean log2 change 0.01, *P = *.97) (Figure 6).

The limited patient population with colonic CD in both cohorts prevented the analysis of anti-integrin αvβ6 concerning the progression of the disease. The logistic regression model for differentiating all CD patients with an indolent disease course from those with an aggressive course demonstrated a poor prognostic performance (AUC = 0.53 [0.42-0.64]) in the discovery cohort (Supplementary Figure 6A). Applying this model to the validation cohort yielded an AUC of 0.60 (0.47-0.74) (Supplementary Figure 6B), with a sensitivity of 68% and a specificity of 50% at its optimal cut-off (57 U_A_/l). Additionally, no associations were observed between antibody positivity or levels and disease course in all patients with CD, regardless of disease location (Supplementary Table 12; Supplementary Figure 7).

## 4. Discussion

This study demonstrated that anti-integrin αvβ6 is a reliable diagnostic and possibly prognostic marker for UC. By conducting an automated in-house EliA assay-based analysis of prospectively collected serum samples from inception, we observed a high diagnostic capacity of the autoantibody to distinguish UC from symptomatic controls. The diagnostic accuracy of anti-integrin αvβ6 outperformed hs-CRP and fcalpro. Adding fcalpro to the anti-integrin αvβ6 model further improved its performance to predict UC. By categorising patients based on their autoantibody status, we found that anti-integrin αvβ6 positivity defines a severe phenotype of UC, characterised by heightened inflammatory activity and high relative levels of IL-17A and GZMB. Furthermore, our study underlines the prognostic significance of the anti-integrin αvβ6 autoantibody, with higher levels at diagnosis indicating a more aggressive trajectory of UC. Crucially, the diagnostic and prognostic utility of the autoantibody was validated in an independent population-based inception cohort from Norway. Incorporating this autoantibody into clinical practice could complement existing markers to evaluate patients with symptoms suggestive of UC and assist clinicians in identifying those with a more severe disease subtype.

Studies have reported increased anti-integrin αvβ6 levels in patients with prevalent UC.^10–13^ Following the initial reports of the presence of the antibody in adult patients with longstanding UC^10,13^ who had undergone various treatments, Livanos et al. examined 2 cohorts of recently diagnosed IBD patients, most of whom were naïve to biologics.^11^ The authors documented a sensitivity for the anti-integrin αvβ6 of 86% and 70% (for 2 independent cohorts) and a specificity of 98% for differentiating UC from non-IBD controls. By examining inception cohorts, we advanced previous knowledge. Specifically, we demonstrated high sensitivity (79%) and specificity (94%) of the autoantibody for differentiating treatment-naïve patients with UC from symptomatic controls in the population-based validation cohort. These results show that anti-integrin αvβ6 has a superior diagnostic performance over previously described serological markers of UC, including atypical perinuclear anti-neutrophil cytoplasmic antibodies.^20^ In clinical translation, our study demonstrated a higher AUC for anti-integrin αvβ6 when compared to hs-CRP and fcalpro. Notably, the accuracy was further augmented by including fcalpro in the analysis of anti-integrin αvβ6. When applied to the validation cohort, the combined anti-integrin αvβ6 and fcalpro model exhibited an AUC of 97%. Consistent with these findings, the reclassification of patients with UC from symptomatic controls improved in both cohorts upon adding the autoantibody to fcalpro.

Moreover, we showed increased autoantibody levels in patients with colonic CD compared to symptomatic controls and healthy controls in the discovery cohort, though this difference did not reach statistical significance in the validation cohort. This is the first study to examine anti-integrin αvβ6 in patients with newly diagnosed colonic CD. The antibodies in question have only been evaluated in a limited group of 16 patients diagnosed with colonic CD in previous studies. No significant disparities in anti-integrin αvβ6 levels were observed when compared to patients with UC.^21^ Numerous studies have established that the genetic profile of colonic CD shows a greater resemblance to UC than to ileal CD.^3^ Findings in transcriptomics and proteomics support this observation, indicating similarities among patients with colonic inflammation in IBD.^5,6^ However, our findings suggest that increased anti-integrin αvβ6 levels are not merely a consequence of colonic inflammation but may reflect a distinct immunological phenotype.

Insights from other disease areas have underscored the importance of autoantibodies, such as anti-citrullinated protein antibodies in rheumatoid arthritis, in diagnostic, and prognostic algorithms,^22^ including predicting outcomes of specific treatments such as rituximab.^23^ Based on these previous findings, we hypothesised that autoantibody seropositive disease would represent a distinct phenotype of UC.

To test this hypothesis, we compared UC patients who were positive for anti-integrin αvβ6 to those who were negative. Our analysis revealed that anti-integrin αvβ6 positive patients exhibited more pronounced inflammatory characteristics in the discovery and validation cohorts. This finding aligns with investigations that have associated anti-integrin αvβ6 levels with extensive (E3) disease^11^ and higher disease activity.^10,13^ However, unlike previous studies, we examined patients with incident IBD, categorising them according to the presence or absence of this autoantibody to advance the interpretation in a clinical setting. In contrast to the differences observed in objective markers of inflammation and disease severity, associations with PROs were less consistent.

Given the proposed role for anti-integrin αvβ6 in epithelial barrier integrity and mucosal homeostasis,^10,11^ we inferred that the serum proteome in patients with UC might vary according to anti-integrin αvβ6 status. Several serum protein markers were differentially regulated between autoantibody positive and negative patients with UC, although only IL-17A and GZMB were confirmed in the validation cohort. The role of IL-17A in IBD has yielded contradictory results.^24,25^ Some studies suggest that IL-17A has a protective role in host defence, contributing to acute immune responses at epithelial and mucosal barriers, promoting tissue repair following injury, and maintaining the integrity of the epithelial tight-junction barrier during inflammation.^24,25^ Nevertheless, excessive IL-17A activation can drive autoimmunity and chronic inflammation, with elevated levels observed in patients with active UC.^26–30^ GZMB is a serine protease predominantly produced by cytotoxic T lymphocytes and natural killer cells, playing a pivotal role in inducing apoptosis in target cells. Kim et al.^31^ showed that GZMB transcripts were overexpressed in CD patients, suggesting it is a potential marker for active CD. Recent studies reported differences in relative protein estimates of GZMB in serum from IBD patients and controls and between patients with UC and CD.^31–33^

Livanos et al.^11^ recently explored the associations between anti-integrin αvβ6 levels and adverse outcomes in 2 cohorts of IBD patients, the majority of whom were naïve to biologics. Elevated levels were associated with increased hazard ratios of a more complicated course of UC. Our study confirmed the association of the autoantibody with disease course, even when applying strict criteria for defining an aggressive disease course. Baseline and dynamic anti-integrin αvβ6 levels were associated with the disease course. Although we cannot exclude any involvement in treatment decisions during this period, we believe this finding supports exploring the autoantibody’s association with specific therapeutic interventions.

This study has several strengths and limitations. A major strength lies in using 2 independent inception IBD cohorts with prospectively collected serum samples, particularly given that the validation cohort was population-based and included only treatment-naïve patients. The comparison of patients with symptoms resembling IBD underscores the diagnostic relevance of the autoantibody. To bolster the advancement of high-throughput method development, we implemented analysis based on the EliA platform. Additionally, the analysis of repeatedly collected samples enabled temporal assessments of anti-integrin αvβ6 levels during the first 3 months after diagnosis. However, the lack of data beyond the first 3-month period limits the interpretation of our results and challenges the possibility of assessing the potential of the autoantibody as a monitoring tool. The inclusion of subsequent follow-up samples in the analysis of associations with protein markers would have provided insights into their stability over time. The absence of ethnicity data in our discovery cohort may affect the generalizability of our findings, even though previous studies from Japan, the United States, and Italy support our findings. Despite our study including more than two-thirds of all participants from previous studies,^10,11,13,21^ the limited number of patients in specific Montreal classification categories hindered meaningful stratified analyses for certain phenotypes. Specifically, the low number of patients with colonic CD prevented us from drawing definite conclusions regarding this subgroup of patients. Interestingly, recent data presented only in abstract format suggest that also patients with colonic CD also exhibit increased levels of the autoantibody.^34^

An additional limitation shared by this study and previous research on adverse disease course outcomes is the lack of standardised criteria to define aggressive disease. To address this, we conducted sensitivity analyses by applying both a more stringent definition and a less strict one. The results were not different when excluding hospital admission from the criteria, whereas the inclusion of patients with UC who start at least 1 targeted therapy within the first year from diagnosis resulted in an improved prognostic performance for the autoantibody model. Because of the prospective nature of our recruitment process, we could not match patients with IBD and symptomatic controls by sex, age, and sampling date. Our evaluation of diagnostic performance only considered hs-CRP and fcalpro, without considering more recently proposed markers for IBD (eg, myeloperoxidase and different lipid species).^17,35^ We cannot exclude the possibility of false negatives resulting from immune exhaustion in some IBD patients or false positives due to cross-reactivity with other integrin family proteins. Additionally, we may have lacked sufficient statistical power to make any firm conclusions from the examined associations with protein markers. For clinical application, extensive registration studies are necessary for regulatory approval and routine clinical implementation. Even though the performance of our prognostic logistic regression model to differentiate between patients with indolent and aggressive UC was modest, our findings suggest that different cut-offs need to be applied depending on the clinical scenario, ie, a diagnostic or a prognostic model.

In conclusion, our study demonstrates the diagnostic and prognostic utility of anti-integrin αvβ6 as a reliable indicator for UC, underscoring its potential for early diagnosis and prediction of clinical outcomes. Through additional validations in diverse cohorts, anti-integrin αvβ6 autoantibodies could significantly contribute to the stratification of UC patients and help identify individuals who would benefit from early intensive treatment to improve long-term outcomes.

## Supplementary Material

jjaf062_suppl_Supplementary_Tables_S1-S12_Figures_S1-S7

## Data Availability

The data underlying this article cannot be shared publicly due to the privacy of the study patients and healthy control participants. However, the data will be made available to other researchers upon reasonable request to the corresponding author.

## Patient and public involvement

Representatives of the Swedish and Norwegian national patient organisations participated in developing the study and its design (Figure 1).
