# Supplementary material for: Anti-integrin αvβ6 IgG antibody as a diagnostic and prognostic marker in ulcerative colitis: A cross-sectional and longitudinal study defining a specific disease phenotype
Source: J Crohns Colitis. 2025 Apr 19;19(5):jjaf062. doi: 10.1093/ecco-jcc/jjaf062 (PMC12086997; doi:10.1093/ecco-jcc/jjaf062)
Supplement: jjaf062_suppl_Supplementary_Tables_S1-S12_Figures_S1-S7 [file jjaf062_suppl_supplementary_tables_s1-s12_figures_s1-s7.docx]

### Supplemental information

**Title: Anti-integrin αvβ6 IgG antibody as a diagnostic and prognostic marker in ulcerative colitis: A cross-sectional and longitudinal study defining a specific disease phenotype.**

**Authors:**

Eleftheria Pertsinidou^1,2^, Benita Salomon^3^, Daniel Bergemalm^4^, Samira Salihovic^3^, Charlotte R.H. Hedin^5,6^, Maria Ling Lundström^7^, Åsa V. Keita^8^, Maria K. Magnusson^9^, Carl Eriksson^4^, May-Bente Bengtson^10^, Olle Grännö^11^, Tone B. Aabrekk^10,12^, Robert Movérare^2,13^, Niclas Rydell^2^, Helena Ekoff^2,7^, Johan Rönnelid^1^, BIO-IBD consortium, Mauro D’Amato^14,15,16^, Trond E. Detlie^12,17^, Gert Huppertz-Hauss^18^, Randi Opheim^18,19^, Petr Ricanek^17,20^, Vendel A. Kristensen^19,21^, Lena Öhman^9^, Johan D. Söderholm^8^, Robert Kruse^3^, Carl M. Lindqvist^3^, Marie Carlson^7^, Dirk Repsilber^3^, Marte L. Høivik^12,19^, Jonas Halfvarson^4^

**Author affiliations:** ^1^Uppsala University, Department of Immunology, Genetics and Pathology, Uppsala, Sweden. ^2^Thermo Fisher Scientific, Uppsala, Sweden. ^3^Örebro University, School of Medical Sciences, Örebro, Sweden. ^4^Örebro University, Department of Gastroenterology, Faculty of Medicine and Health, Örebro, Sweden. ^5^Karolinska Institutet, Department of Medicine Solna, Stockholm, Sweden. ^6^Karolinska University Hospital, Centre for Digestive Health, Department of Gastroenterology, Dermatovenereology and Rheumatology, Stockholm, Sweden. ^7^Uppsala University, Department of Medical Sciences: Gastroenterology and Hepatology, Uppsala, Sweden. ^8^Linköping University, Department of Biomedical and Clinical Sciences, Linköping, Sweden. ^9^University of Gothenburg, Sahlgrenska Academy, Department of Microbiology and Immunology, Institute of Biomedicine, Gothenburg, Sweden. ^10^Vestfold Hospital Trust, Department of Gastroenterology, Tønsberg, Norway. ^11^Örebro University, Department of Laboratory Medicine, Clinical Microbiology, Faculty of Medicine and Health, Örebro, Sweden ^12^University of Oslo, Institute of Clinical Medicine, Oslo, Norway. ^13^Uppsala University, Department of Medical Sciences: Respiratory, Allergy and Sleep Research, Uppsala, Sweden. ^14^Gastrointestinal Genetics Lab, CIC bioGUNE - BRTA, Derio, Spain. ^15^Ikerbasque, Basque Foundation for Science, Bilbao, Spain. ^16^Department of Medicine and Surgery, LUM University, Casamassima, Italy. ^17^Akershus University Hospital, Department of Gastroenterology, Lørenskog, Norway. ^18^University of Oslo, Institute of Health and Society, Oslo, Norway. ^19^Oslo University Hospital, Department of Gastroenterology, Oslo, Norway. ^20^Lovisenberg Diaconal Hospital, Department of Gastroenterology, Oslo, Norway. ^21^Lovisenberg Diaconal Hospital, Unger-Vetlesen Institute, Oslo, Norway.

**Collaborators:**

**Sven Almer**, [sven.almer@ki.se](mailto:sven.almer@ki.se)

Karolinska Institutet, Department of Medicine Solna, Stockholm, Sweden; Karolinska University Hospital, Center for Digestive Health, Department of Gastroenterology, Dermatovenereology and Rheumatology, Stockholm, Sweden.

**Hans Strid**, [hans.strid@regionstockholm.se](mailto:hans.strid@regionstockholm.se)

Karolinska University Hospital, Centre for Digestive Health, Department of Gastroenterology, Dermatovenereology and Rheumatology, Stockholm, Sweden.

**Henrik Hjortswang**, [Henrik.Hjortswang@regionostergotland.se](mailto:Henrik.Hjortswang@regionostergotland.se)

Department of Gastroenterology and Hepatology, Linköping University, Linköping, Department of Health, Medicine, and Caring Sciences, Linköping University, Linköping, Sweden.

**Francesca Bresso**, [francesca.bresso@regionstockholm.se](mailto:francesca.bresso@regionstockholm.se)

Department of Medicine, Karolinska Institutet, Solna, and Division of Gastroenterology, Karolinska University Hospital, Stockholm, Sweden.

**Johann Hreinsson**,  [johann.hreinsson@vgregion.se](mailto:johann.hreinsson@vgregion.se)

Department of Molecular and Clinical Medicine, Institute of Medicine, Sahlgrenska Academy, University of Gothenburg, Gothenburg, Sweden.

**André Blomberg**[andre.blomberg@vgregion.se](mailto:andre.blomberg@vgregion.se)

Department of Medicine, Geriatrics and Emergency Medicine, Sahlgrenska University Hospital, Östra Hospital, Gothenburg, Sweden.

**Adam Carstens**, [Adam.Carstens@erstadiakoni.se](mailto:Adam.Carstens@erstadiakoni.se)

Department of Internal Medicine, Ersta Hospital, Stockholm, Sweden.

****Correspondence:***

*Jonas Halfvarson, Professor, Department of Gastroenterology,*

*Faculty of Medicine and Health, Örebro University, Örebro, Sweden.*

*Phone: +46 19 303000, +46 738 082361*

*Email: jonas.halfvarson@regionorebrolan.se*

### Inventory of supporting information

Table of Contents

Supplementary Methods

Supplementary Tables

Supplementary Table 1 6

Supplementary Table 2 7

Supplementary Table 3 8

Supplementary Table 4 9

Supplementary Table 5 10

Supplementary Table 6 11

Supplementary Table 7 12

Supplementary Table 8 13

Supplementary Table 9 14

Supplementary Table 10 14

Supplementary Table 11 15

Supplementary Table 12 15

Supplementary Figures

Supplementary Figure 1 16

Supplementary Figure 2 16

Supplementary Figure 3 17

Supplementary Figure 4 18

Supplementary Figure 5 18

Supplementary Figure 6 19

Supplementary Figure 7 19

## Discovery and validation cohorts

Patients with gastrointestinal symptoms, such as diarrhoea, abdominal pain, and blood or mucus in stool, indicative of IBD, were included in the discovery cohort. Exclusion criteria were a previous IBD diagnosis, treatment with IBD-related medications, including 5-ASA, sulfasalazine, corticosteroids, immunomodulators, biologics, Janus kinase-inhibitors, and sphingosine-1-phosphate receptor modulators, and inability to provide consent. After obtaining informed written consent, serum and stool samples were collected, and patient-reported outcome (PRO) measures were recorded. Patients diagnosed with IBD were categorised according to their clinical phenotype using the Montreal classification.^1^ The endoscopic Mayo Clinic subscore was applied to determine endoscopic activity in patients with UC, whereas ulcers were recorded in patients with CD. Clinical disease activity was defined based on the Harvey Bradshaw Index (HBI) for CD and the partial Mayo Clinic score for UC. Patients were prospectively followed according to clinical practice and re-sampled during follow-up.

The inclusion criteria of the validation cohort were analogous to those of the discovery cohort, and patients were followed prospectively. Patients with symptoms of IBD but with no inflammation at endoscopic or histologic examinations were included as symptomatic non-IBD controls.

Setting

The healthcare system in Sweden and Norway is tax-funded, and access to care is universal. The personal identification number assigned to all permanent residents in each country enables healthcare to maintain contact with patients over time with almost no loss of follow-up. Patients with suspected IBD are typically referred to a gastroenterologist or internist in a hospital-based outpatient facility for clinical work-up, including endoscopy and potential imaging.

Protein analysis and preprocessing

Normalised Protein eXpression (NPX) values were obtained, levels below the limit of detection (LOD) were reported, and quality control was performed according to the manufacturer’s protocol. Proteins and samples with more than 50% of values below LOD or with a quality warning were excluded (22 proteins and 43 samples across both cohorts). The remaining values below LOD were replaced with the LOD value of the respective protein. Mean values were used for proteins that remained after the quality control and were present on both panels (VEGFA, TRAIL, SCF, TGF-alpha, HGF). Following quality control, missing NPX values (SIC-IBD, n=19 and IBSEN III, n=33) were imputed using the k-nearest neighbour algorithm. ComBat was used to adjust for batch effects^2^ and principal component analysis to control for outliers.

Supplementary Table 1. Rates of anti-integrin αvβ6 positivity per disease group in the discovery and validation inception cohorts.

|  | DISCOVERY  COHORT | VALIDATION  COHORT |
| --- | --- | --- |
|  | **Anti-integrin αvβ6 positives, N (%)** | |
| Ulcerative colitis | 133/183 (73) | 163/206 (79) |
| Ileal Crohn’s disease (L1) | 2/42 (5) | 4/54 (7) |
| Colonic Crohn’s disease (L2) | 19/40 (48) | 6/22 (27) |
| Ileocolonic Crohn’s disease (L3) | 6/28 (21) | 6/35 (17) |
| IBD-U | 18/23 (78) | 10/21 (48) |
| Symptomatic controls | 10/146 (7) | 12/204 (6) |
| Healthy controls | 4/48 (8) | NA |

Abbreviations: IBD-U, IBD-unclassified.

Supplementary Table 2. Net reclassification and integrated discrimination improvement of ulcerative colitis with hs-CRP and with and without anti-integrin αvβ6 in the discovery cohort, using the prevalence of UC (56%).

|  | No. of patients | | | Reclassified (%) | | |
| --- | --- | --- | --- | --- | --- | --- |
| hs-CRP | hs-CRP + anti-integrin αvβ6 | | |  |  |  |
|  | < 56% | ≥ 56% | Total | Downward (%) | Upward (%) | Net reclassified (%) |
| Cases with UC |  |  |  |  |  |  |
| < 56% | 20 | 65 | 85 | 11% | 36% | 25% |
| ≥ 56% | 20 | 78 | 98 |  |  |  |
| Total | 40 | 143 | 183 |  |  |  |
| Symptomatic controls |  |  |  |  |  |  |
| < 56% | 88 | 6 | 94 | 32% | 4% | -28% |
| ≥ 56% | 47 | 5 | 52 |  |  |  |
| Total | 135 | 11 | 146 |  |  |  |
| NRI (SE, *P-*value) | 0.52 (0.07, *P* < 0.001) | | | | | |
| IDI (SE, *P*-value) | 0.48 (0.02, *P* < 0.001) | | | | | |

Abbreviations: hs-CRP, high sensitivity C-reactive protein; UC, Ulcerative colitis; NRI, net reclassification index; IDI, integrated discrimination index; SE, standard error

Supplementary Table 3. Net reclassification and integrated discrimination improvement of ulcerative colitis with fcalpro and with and without anti-integrin αvβ6 in the discovery cohort, using the prevalence of UC (56%).

|  | No. of patients | | | Reclassified (%) | | |  |
| --- | --- | --- | --- | --- | --- | --- | --- |
| fcalpro | fcalpro + anti-integrin αvβ6 | | |  |  |  |  |
|  | < 56% | ≥ 56% | Total | Downward (%) | Upward (%) | Net reclassified (%) |  |
| Cases with UC |  |  |  |  |  |  |  |
| < 56% | 14 | 30 | 44 | 9% | 21% | 12% |  |
| ≥ 56% | 13 | 84 | 97 |  |  |  |  |
| Total | 27 | 114 | 141 |  |  |  |  |
| Symptomatic controls |  |  |  |  |  |  |  |
| < 56% | 70 | 3 | 73 | 24% | 3% | -21% |  |
| ≥ 56% | 25 | 7 | 32 |  |  |  |  |
| Total | 95 | 10 | 105 |  |  |  |  |
| NRI (SE, *P*-value) | 0.33 (0.07, *P* < 0.001) | | | | | | |
| IDI (SE, *P*-value) | 0.30 (0.03, *P* < 0.001) | | | | | | |

Abbreviations: fcalpro, faecal calprotectin; UC, Ulcerative colitis; NRI, net reclassification index; IDI, integrated discrimination index; SE, standard error

Supplementary Table 4. Net reclassification and integrated discrimination improvement of ulcerative colitis with hs-CRP and with and without anti-integrin αvβ6 in the validation cohort, using the prevalence of UC (50%).

|  | No. of patients | | | Reclassified (%) | | | |
| --- | --- | --- | --- | --- | --- | --- | --- |
| hs-CRP | hs-CRP + anti-integrin αvβ6 | | |  |  |  | |
|  | < 50% | ≥ 50% | Total | Downward (%) | Upward (%) | | Net reclassified (%) |
| Cases with UC |  |  |  |  |  | |  |
| < 50% | 21 | 72 | 93 | 6% | 35% | | 29% |
| ≥ 50% | 13 | 97 | 110 |  |  |  |  |
| Total | 34 | 169 | 203 |  |  |  |  |
| Symptomatic controls |  |  |  |  |  | |  |
| < 50% | 127 | 11 | 138 | 26% | 5% | | -21% |
| ≥ 50% | 53 | 11 | 64 |  |  |  |  |
| Total | 180 | 22 | 202 |  |  |  |  |
| NRI (SE, *P*-value) | 0.49 (0.06, *P* < 0.001) | | | | | | |
| IDI (SE, *P*-value) | 0.53 (0.02, *P* < 0.001) | | | | | | |

Abbreviations: hs-CRP, high sensitivity C-reactive protein; UC, Ulcerative colitis; NRI, net reclassification index; IDI, integrated discrimination index; SE, standard error

Supplementary Table 5. Net reclassification and integrated discrimination improvement of ulcerative colitis with fcalpro and with and without anti-integrin αvβ6 in the validation cohort, using the prevalence of UC (50%).

|  | Number of patients | | | Reclassified (%) | | |  |
| --- | --- | --- | --- | --- | --- | --- | --- |
| fcalpro | fcalpro + anti-integrin αvβ6 | | |  |  |  |  |
|  | <50% | ≥50% | Total | Downward (%) | Upward (%) | Net reclassified (%) |  |
| Cases with UC |  |  |  |  |  |  |  |
| <50% | 10 | 26 | 36 | 6% | 16% | 11% |  |
| ≥50% | 9 | 116 | 125 |  |  |  |  |
| Total | 19 | 142 | 161 |  |  |  |  |
| Symptomatic controls |  |  |  |  |  |  |  |
| <50% | 118 | 4 | 122 | 13% | 3% | -11% |  |
| ≥50% | 20 | 9 | 29 |  |  |  |  |
| Total | 138 | 13 | 151 |  |  |  |  |
| NRI (SE, *P*-value) | 0.21 (0.05, *P<*0.001) | | | | | | |
| IDI (SE, *P*-value) | 0.30 (0.03, *P*<0.001) | | | | | | |

Abbreviations: fcalpro, faecal calprotectin; UC, ulcerative colitis; NRI, net reclassification index; IDI, integrated discrimination index; SE, standard error

Supplementary Table 6. Demographic characteristics of UC patients in the discovery (N=183) and validation (N=206) cohort based on anti-integrin αvβ6 status.

|  | DISCOVERY  SIC-IBD | | | VALIDATION  IBSEN III | | |
| --- | --- | --- | --- | --- | --- | --- |
|  | **Anti-integrin αvβ6 positives** | **Anti-integrin αvβ6 negatives** | ***P*-value** | **Anti-integrin αvβ6 positives** | **Anti-integrin αvβ6 negatives** | ***P-*value** |
| Median (IQR) Age | 34 (26-46) | 32 (25-46) | 0.65 | 32 (24-44) | 39 (27-54) | **0.01** |
| Male, N (%) | 82 (62) | 25 (50) | 0.15 | 96 (59) | 23 (53) | 0.52 |
| Smoking, N (%) |  |  |  |  |  |  |
| Never | 66 (58) | 20 (47) | 0.17 | 78 (58) | 15 (48) | 0.41 |
| Former | 40 (35) | 16 (37) |  | 51 (38) | 13 (42) |  |
| Active | 8 (7) | 7 (16) |  | 6 (4) | 3 (10) |  |

Statistical analyses were conducted using the χ^2^ and Mann-Whitney U tests for continuous parameters. Information was missing for Smoking (n=26) in SIC-IBD and Smoking (n=40) in IBSEN III. Abbreviations: IQR, interquartile range; UC, ulcerative colitis.

Supplementary Table 7. Demographic characteristics of patients with colonic Crohn’s disease in the discovery (N=40) and validation (N=22) cohort based on anti-integrin αvβ6 status.

|  | DISCOVERY  SIC-IBD | | | VALIDATION  IBSEN III | | |
| --- | --- | --- | --- | --- | --- | --- |
|  | **Anti-integrin αvβ6 positives** | **Anti-integrin αvβ6 negatives** | ***P-*value** | **Anti-integrin αvβ6 positives** | **Anti-integrin αvβ6 negatives** | ***P*-value** |
| Median (IQR) Age | 31 (27-54) | 32 (27-58.5) | 0.97 | 37 (24-59.5) | 35.5 (25.5-53.3) | 0.68 |
| Male, N (%) | 9 (50) | 11 (50) | 0.75 | 1 (17) | 5 (31) | 0.49 |
| Smoking, N (%) |  |  |  |  |  |  |
| Never | 9 (60) | 13 (68) | 0.88 | 5 (83) | 6 (50) | 0.56 |
| Former | 4 (27) | 4 (21) |  | 1 (17) | 5 (42) |  |
| Active | 2 (13) | 2 (11) |  | 0 | 1 (8) |  |

Statistical analyses were conducted using the χ2 or Fisher’s exact test (for expected frequencies <5) and, for continuous parameters, the Mann-Whitney U test. Information was missing for Smoking n=6 in SIC-IBD and Smoking n=4 in IBSEN III. Abbreviations: IQR, interquartile range.

Supplementary Table 8. Associations of anti-integrin αvβ6 with clinical parameters and patient-reported outcomes among patients with colonic Crohn’s disease (N=40) in the discovery cohort.

|  | Anti-integrin αvβ6 positives (N=19) | Anti-integrin αvβ6 negatives (N=21) | *P-*value |
| --- | --- | --- | --- |
| CD Behaviour, N (%) |  |  |  |
| B1 | 17 (94) | 17 (85) | 0.74 |
| B2 | 1 (6) | 1 (5) |  |
| B3 | 0 | 2 (10) |  |
| Median (IQR) HBI | 4 (2.5-7) | 3.5 (1.8-9.5) | 1 |
| Median (IQR) hs-CRP | 6.5 (1.9-52) | 11 (2.3-46.5) | 0.71 |
| Median (IQR) faecal calprotectin (μg/g) | 875.5 (498.3-1980) | 475 (246-1420) | 0.17 |
| Median (IQR) Albumin (g/L) | 35 (30-37) | 38 (32-41.5) | 0.06 |
| Median (IQR) Number of liquid/soft stools | 2 (0-5) | 2 (0-5.8) | 0.98 |
| Abdominal pain, N (%) |  |  |  |
| None | 3 (21) | 3 (18) | 0.77 |
| Mild | 4 (29) | 8 (47) |  |
| Moderate | 5 (36) | 5 (29) |  |
| Severe | 2 (14) | 1 (6) |  |

Statistical analyses were conducted using the χ2 test or Fisher’s exact test (for expected frequencies <5) and, for continuous parameters, the Mann-Whitney U test. Information was missing for CD behaviour n=2, HBI n=25, faecal calprotectin n=9, Number of liquid/soft stools n=13, abdominal pain n=9. Abbreviations: CD, Crohn’s disease; IQR, interquartile range; HBI, Harvey Bradshaw Index; hs-CRP, high sensitivity C-reactive protein.

Supplementary Table 9. Associations between anti-integrin αvβ6, clinical parameters, and patient-reported outcomes among patients with colonic Crohn’s disease (N=22) in the validation cohort.

|  | Anti-integrin αvβ6 positives (N=6) | Anti-integrin αvβ6 negatives (N=16) | *P-*value |
| --- | --- | --- | --- |
| CD Behaviour, N (%) |  |  |  |
| B1 | 6 (100) | 15 (94) | 0.53 |
| B2 | 0 | 1 (6) |  |
| Median (IQR) HBI | 2 (0-6.3) | 6 (5-8) | 0.08 |
| Median (IQR) CRP | 2.3 (1-20.7) | 2.4 (1.1-21) | 0.88 |
| Median (IQR) faecal calprotectin (μg/g) | 447 (192.5-1801) | 298.5 (150.8-968.5) | 0.49 |
| Median (IQR) Number of liquid/soft stools | 1 (0-3.8) | 3.5 (2-4.8) | 0.13 |
| Abdominal pain, N (%) |  |  |  |
| None | 4 (67) | 3 (19) | 0.08 |
| Mild | 1 (17) | 10 (63) |  |
| Moderate | 1 (17) | 3 (19) |  |
| Presence of ulcers at endoscopy, N (%) | 5 (100) | 13 (93) | 1.0 |

Statistical analyses were conducted using the χ2 test or Fisher’s exact test (for expected frequencies <5) and, for continuous parameters, the Mann-Whitney U test. Information was missing for faecal calprotectin n=5 and Presence of ulcers at endoscopy n=3. Abbreviations: CD, Crohn’s disease; IQR, interquartile range; HBI, Harvey Bradshaw Index; hs-CRP, high sensitivity C-reactive protein.

Supplementary Table 10. Log2 fold change (FC) values of the significantly dysregulated proteins in UC patients in the validation and discovery cohorts.

|  | DISCOVERY  SIC-IBD | | VALIDATION  IBSEN III | |
| --- | --- | --- | --- | --- |
|  | **Log2FC** | **Raw *P*-value** | **Log2FC** | **Raw *P*-value** |
| SYND1 | 0.59 | < 0.0001 | 0.18 | 0.081 |
| IL17A | 0.96 | < 0.0001 | 0.47 | 0.005 |
| GZMB | 1.03 | < 0.0001 | 0.84 | 0.001 |
| MMP1 | 0.55 | 0.001 | -0.18 | 0.11 |
| CXCL13 | 0.33 | 0.001 | 0.17 | 0.132 |

Abbreviations: GZMB, Granzyme B; SYND1, Syndecan 1; MMP-1, Matrix Metalloproteinase 1; IL-17A, Interleukin 17A; CXCL13, Chemokine Ligand 13.

Supplementary Table 11. Associations between anti-integrin αvβ6 and disease course in UC patients in the validation and discovery cohorts.

|  | DISCOVERY  SIC-IBD | | | VALIDATION  IBSEN III | | |
| --- | --- | --- | --- | --- | --- | --- |
|  | **Anti-integrin αvβ6 positives** | **Anti-integrin αvβ6 negatives** | ***P-*value** | **Anti-integrin αvβ6 positives** | **Anti-integrin αvβ6 negatives** | ***P-*value** |
| Disease outcome, N (%) |  |  |  |  |  |  |
| Indolent course | 74 (67) | 52 (88) | **0.003** | 109 (89) | 65 (94) | 0.26 |
| Aggressive course | 36 (33) | 7 (12) |  | 13 (11) | 4 (6) |  |

Statistical analyses were conducted using the χ^2^ test. Information was missing for n=14 in SIC-IBD and n=15 in IBSENIII. The disease course was based on a composite outcome of colectomy, hospital admission for active disease, treatment refractoriness towards ≥2 biological agents, and the use of >2 courses of corticosteroids or a cumulative corticosteroid dose of >2.5 g.

Supplementary Table 12. Associations between anti-integrin αvβ6 and disease course in CD patients in the validation and discovery cohorts.

|  | DISCOVERY  SIC-IBD | | | VALIDATION  IBSEN III | | |
| --- | --- | --- | --- | --- | --- | --- |
|  | **Anti-integrin αvβ6 positives** | **Anti-integrin αvβ6 negatives** | ***P*-value** | **Anti-integrin αvβ6 positives** | **Anti-integrin αvβ6 negatives** | ***P*-value** |
| Disease outcome, N (%) |  |  |  |  |  |  |
| Indolent course | 35 (51) | 26 (63) | 0.2 | 50 (79) | 51 (89) | 0.13 |
| Aggressive course | 34 (49) | 15 (37) |  | 13 (21) | 6 (11) |  |

Statistical analyses were conducted using the χ^2^ test. Information was missing for n=11 in SIC-IBD and n=19 in IBSEN III. The disease course was based on a composite outcome of colectomy, hospital admission for active disease, treatment refractoriness towards ≥2 biological agents, and the use of >2 courses of corticosteroids or a cumulative corticosteroid dose of >2.5 g.

**
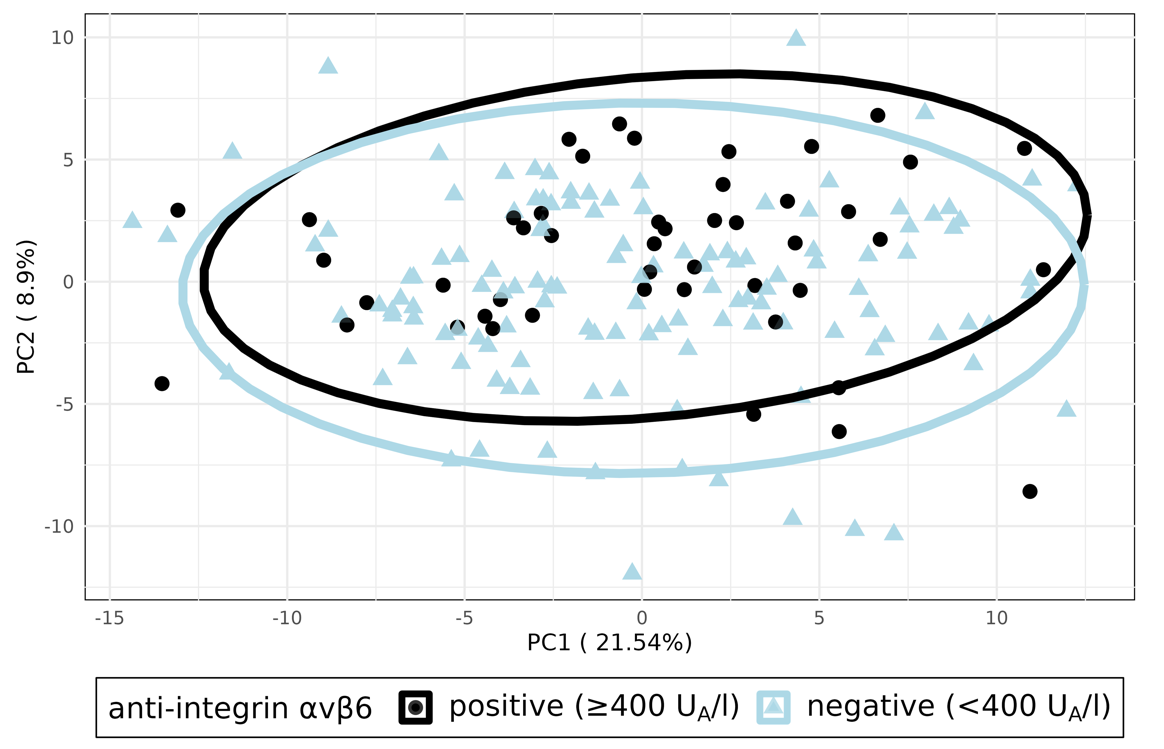
**

Supplementary Figure 1. Principal component analysis of the overall protein profile by anti-integrin αvβ6 status in ulcerative colitis patients in the discovery cohort (N=173).


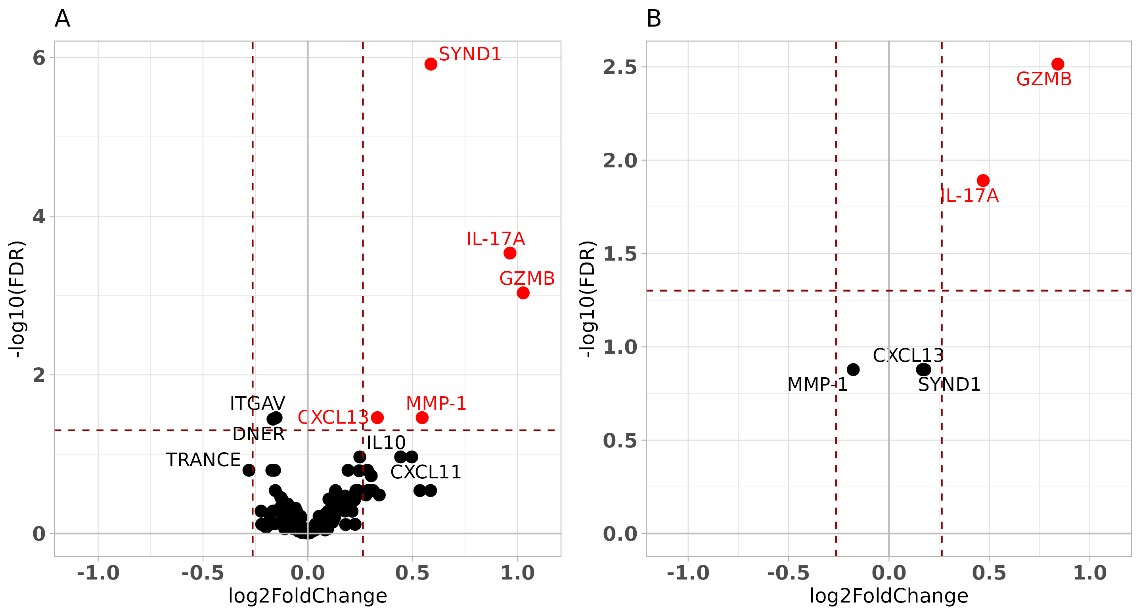


Supplementary Figure 2. Volcano plots visualising serum proteins with significantly different relative levels in patients with ulcerative colitis classified as anti-integrin positive (≥400 U_A_/l) compared to patients classified as anti-integrin negative (<400 U_A_/l) in A) SIC-IBD, anti-integrin positives N=126 vs anti-integrin negatives N=47 and B) IBSEN III, anti-integrin positives N=160 vs anti-integrin negatives N=41. Relative protein levels were compared using Welch’s t-test. False discovery rate (FDR) values were calculated using the Benjamini-Hochberg procedure to adjust for multiple testing (q-value). A q-value cutoff of <0.05 was chosen and a fold change of >1.2. Proteins with significantly higher relative levels of anti-integrin positives are highlighted in red. Abbreviations: GZMB, Granzyme B; SYND1, Syndecan 1; MMP-1, Matrix Metalloproteinase 1; IL-17A, Interleukin 17A; CXCL13, Chemokine Ligand 13.


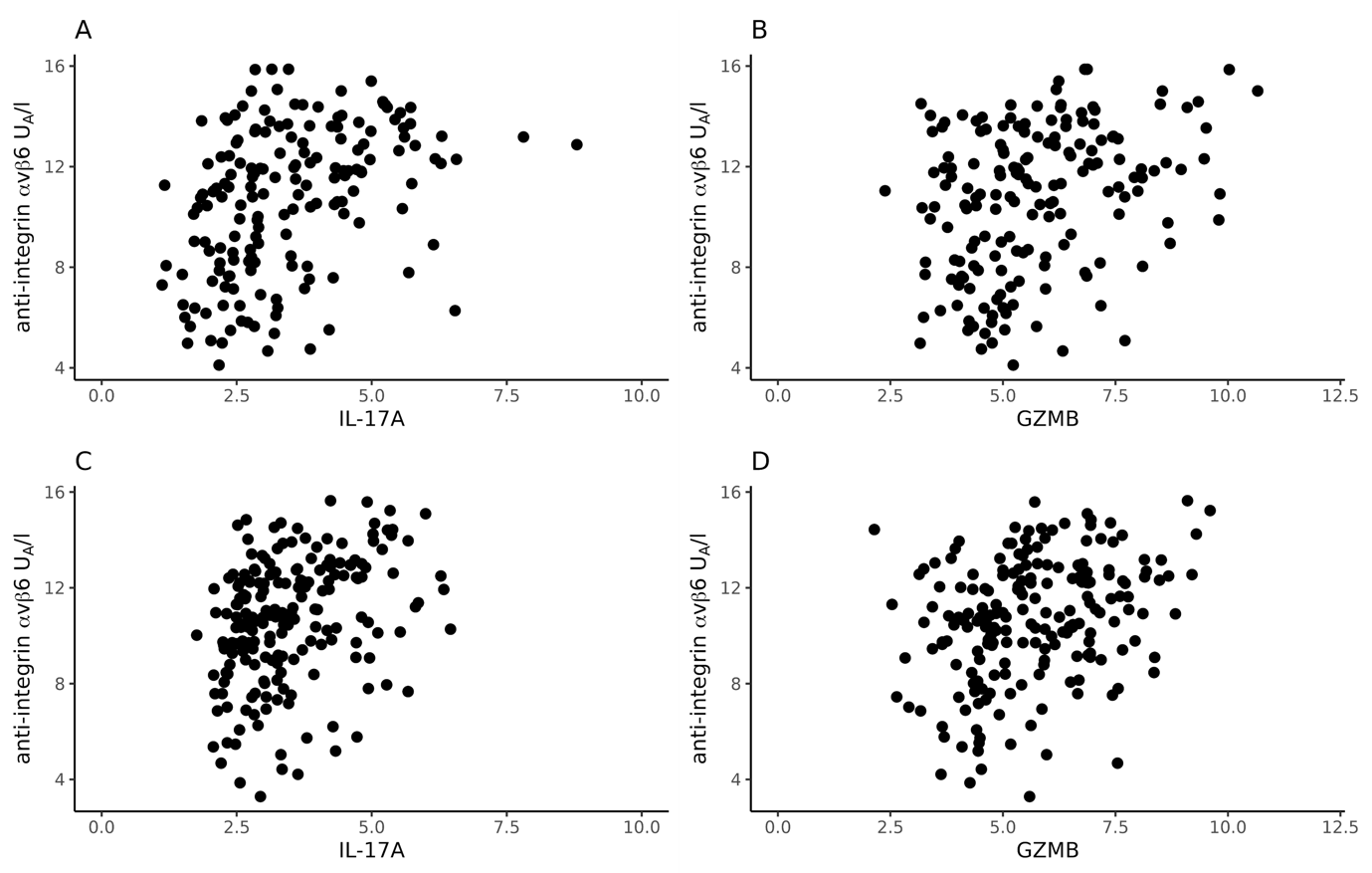


Supplementary Figure 3. Pearson correlation between anti-integrin αvβ6 and the two significantly dysregulated proteins, IL-17A and GZMB. A) IL-17A in SIC-IBD; Correlation coefficient (r) =0.39; q-value <0.0001, B) GZMB in SIC-IBD; r=0.33; q-value <0.001, C) IL-17A in IBSEN III; r=0.34, q-value <0.0001, D) GZMB in IBSEN III; r=0.31 q-value <0.001. Log2 scale is shown for all the variables. Abbreviations: GZMB, Granzyme B; IL-17A, Interleukin 17A; FDR, False discovery rate; UC, ulcerative colitis.

Supplementary Figure 4. Using logistic regression, the receiver operating characteristic (ROC) curves illustrate the prognostic performance of aggressive vs indolent disease course in UC patients in the discovery and validation cohorts. The model performance and validity measures were as follows for anti-integrin αvβ6: (A) in the discovery cohort AUC (95% CI) 0.62 (0.52-0.72), (B) in the validation cohort AUC (95% CI) 0.61 (0.48-0.75). An aggressive course of UC was defined as colectomy, hospital admission for active disease, unresponsiveness to ≥2 advanced therapies (i.e., biologics or JAK inhibitors), or extensive use of corticosteroids. Abbreviations: CI, confidence interval

Supplementary Figure 5. Using logistic regression, the receiver operating characteristic (ROC) curves illustrate the prognostic performance of aggressive vs indolent disease course in UC patients in the validation cohorts. The model performance and validity measures were as follows for anti-integrin αvβ6: AUC (95% CI) 0.72 (0.62-0.82). An aggressive course of UC was defined as colectomy, hospital admission for active disease, start of one targeted therapy (i.e., biologics or JAK inhibitors) within the first year from diagnosis, or extensive use of corticosteroids. Abbreviations: CI, confidence interval

A

B

Supplementary Figure 6. Using logistic regression, the receiver operating characteristic (ROC) curves illustrate the prognostic performance of aggressive vs indolent disease course in CD patients in the discovery and validation cohorts. The model performance and validity measures were as follows for anti-integrin αvβ6: (A) in the discovery cohort AUC (95% CI) 0.53 (0.42-0.64), (B) in the validation cohort AUC (95% CI) 0.60 (0.47-0.74). An aggressive course of CD was defined as colectomy, hospital admission for active disease, unresponsiveness to ≥2 advanced therapies (i.e., biologics or JAK inhibitors), or extensive use of corticosteroids. Abbreviations: CI, confidence interval


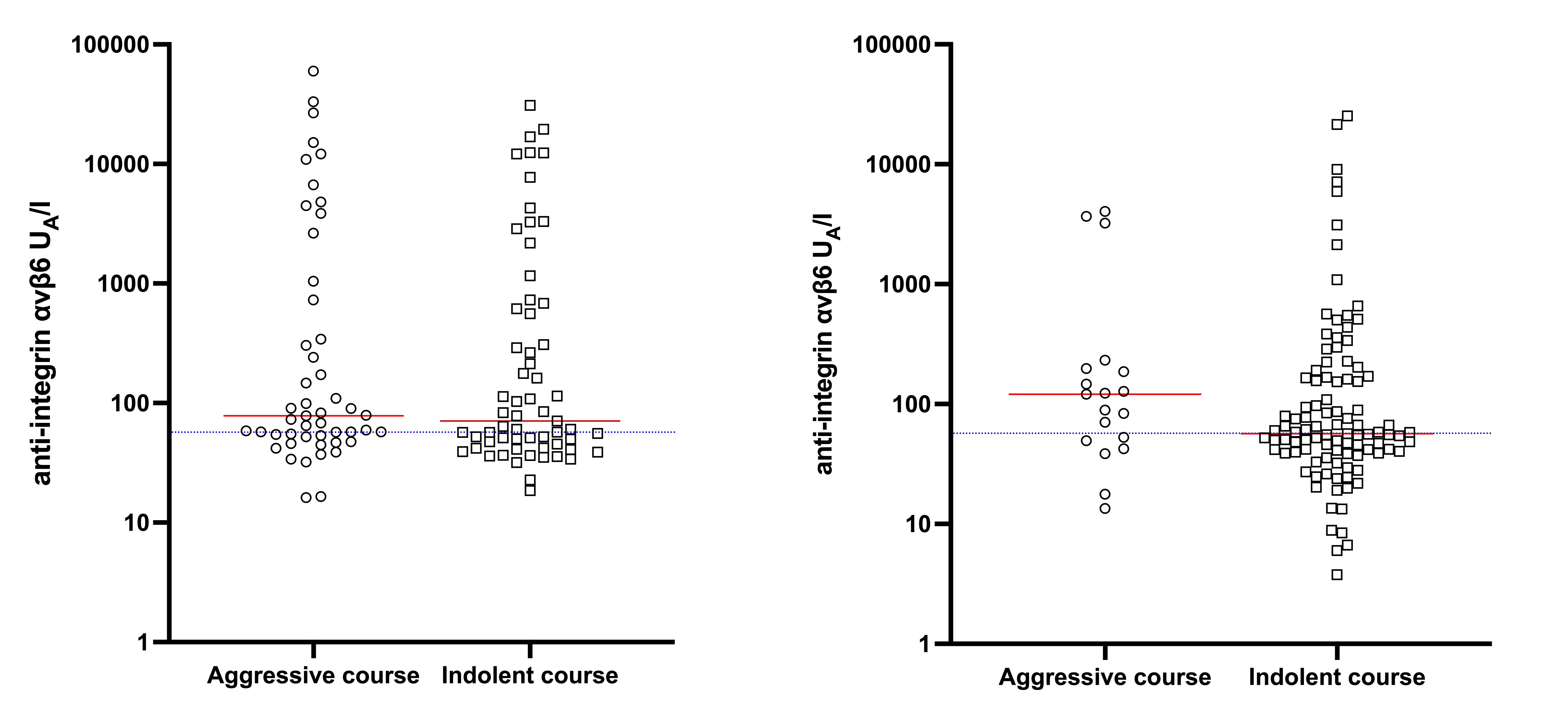


A

B

Supplementary Figure 7. Anti-integrin αvβ6 levels by disease course in A) CD patients in SIC-IBD and B) CD patients in IBSEN III. The red line represents the median, and the blue dotted line is set to the suggested cut-off (57 U_A_/l). The disease course was based on a composite outcome of colectomy, hospital admission for active disease, treatment refractoriness towards ≥2 biological agents, and the use of >2 courses of corticosteroids or a cumulative corticosteroid dose of >2.5 g.

## References

1. Silverberg, M. S., Satsangi, J., Ahmad, T., Arnott, I. D., Bernstein, C. N., Brant, S. R. et al. Toward an integrated clinical, molecular and serological classification of inflammatory bowel disease: report of a Working Party of the 2005 Montreal World Congress of Gastroenterology. *Can J Gastroenterol* 2005; **19 Suppl A**: 5A-36A.

2. Johnson, W. E., Li, C. and Rabinovic, A. Adjusting batch effects in microarray expression data using empirical Bayes methods. *Biostatistics* 2007; **8**: 118-127.
